# Supplementary material for: Growth, Evapotranspiration, Gas Exchange and Chl a Fluorescence of Ipê-Rosa Seedlings at Different Levels of Water Replacement
Source: Plants (Basel). 2024 Oct 11;13(20):2850. doi: 10.3390/plants13202850 (PMC11511352; doi:10.3390/plants13202850)
Supplement: Supplementary file 1 [file plants-13-02850-s001.zip › plants-3192723-supplementary.pdf]

Supplementary Table S1. Adjusted equations for non-destructive growth variables of ipê-rosa seedlings at different levels of water replacement (LWR), as a function of accumulated degree days (DDA), in a greenhouse (linked to the curves in Figure 5; in this case, linear equation ($y = B0 + B1\ DDA$) or quadratic equation ($y = B0 + B1\ DDA + B1\ DDA^2$).

**Regression Fit**						
Height (cm)						
LWR	B0	B1	B2	R^2	p-value	Fit
25%	3.6731	0.0040	-	0.9404	< 0.0001	Linear
50%	-7.5659	0.0230	-	0.9399	< 0.0001	Linear
75%	-15.7444	0.0370	-	0.9387	< 0.0001	Linear
100%	-19.8946	0.0441	-	0.9529	< 0.0001	Linear
Basal stem diameter (mm)						
LWR	B0	B1	B2	R^2	p-value	Fit
25%	0.8981	0.0019	-	0.9315	< 0.0001	Linear
50%	-2.6150	0.0084	-	0.9848	< 0.0001	Linear
75%	-3.8027	0.0111	-	0.9925	< 0.0001	Linear
100%	-4.3303	0.0124	-	0.9950	< 0.0001	Linear
Number of leaves						
LWR	B0	B1	B2	R^2	p-value	Fit
25%	6.0418	-0.0031	3.00E-06	0.9835	< 0.0001	Quadratic
50%	0.1498	0.0108	-	0.9991	< 0.0001	Linear
75%	0.0777	0.0120	-	0.9949	< 0.0001	Linear
100%	-4.9563	0.0237	-6.00E-06	0.9979	< 0.0001	Quadratic
Number of leaflets						
LWR	B0	B1	B2	R^2	p-value	Fit
25%	7.9715	-0.0083	6.00E-06	0.9646	< 0.0001	Quadratic
50%	16.1861	-0.0362	3.10E-05	0.9985	< 0.0001	Quadratic
75%	5.8707	-0.0146	2.60E-05	0.9897	< 0.0001	Quadratic
100%	-2.3092	0.0035	2.00E-05	0.9869	< 0.0001	Quadratic

R^2: coefficient of determination; p-value: statistical significance (p-value - <0.0001 implies the significance of the fit at a level below 0.01% probability).

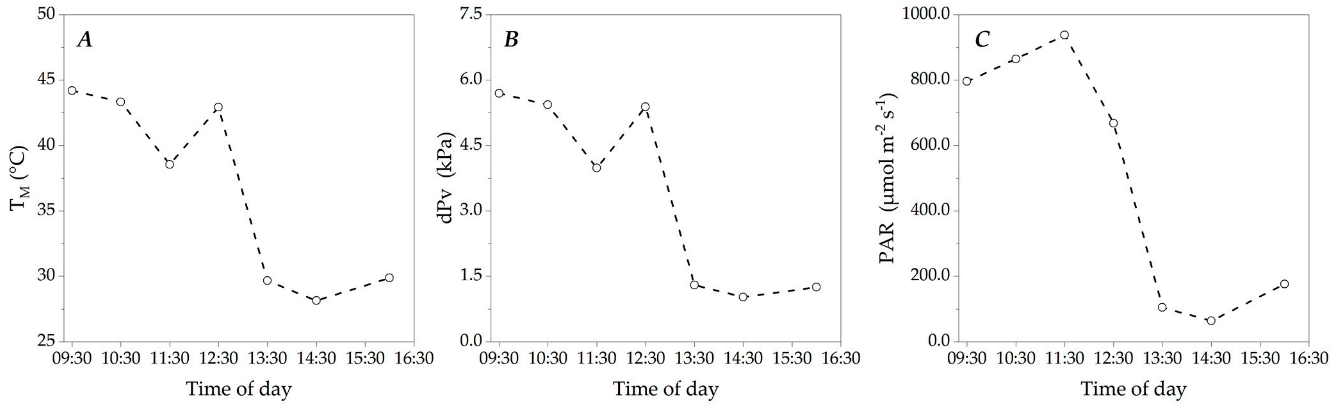

Supplementary Figure S1. Hourly variations of mean air temperature – T_M (A), water vapor pressure deficit – dPv (B) and photosynthetically active radiation – PAR (C), at 100 DAT (1558 DDA), in a greenhouse (internal measurements), Sinop City, Mato Grosso state, Brazil.
